# Tractography-Based Asymmetries in Acquired Brain Injury: Contributions to the Neuropsychological Profile and Rehabilitation in a Case-Series

**DOI:** 10.3390/brainsci15111155

**Published:** 2025-10-28

**Authors:** Rosario Bordón Guerra, Wenceslao Peñate Castro, Eilin Ferreiro Díaz-Velis, Coralia Sosa Pérez, Sara Bisshopp Alfonso, María Hernández Hernández, José Luis Hernández Fleta, Jesús Morera Molina

**Affiliations:** 1 Department of Psychiatry, Hospital Universitario de Gran Canaria Doctor Negrín, 35010 Las Palmas de Gran Canaria, Spain; 2 Department of Medical and Surgical Sciences, University of Las Palmas de Gran Canaria, 35001 Las Palmas de Gran Canaria, Spain; 3 Department of Clinical Psychology, Psychobiology and Methodology, Faculty of Psychology, University of La Laguna, 38200 San Cristóbal de La Laguna, Spain; 4 Department of Neurosurgery, Hospital Universitario de Gran Canaria Doctor Negrín, 35010 Las Palmas de Gran Canaria, Spain; 5 Department of Medical and Surgical Sciences, Psychiatry and Medical Psychology Knowledge Area, University of Las Palmas de Gran Canaria, 35001 Las Palmas de Gran Canaria, Spain; jherfle@gobiernodecanarias.org

**Keywords:** acquired brain injury, diffusion tensor imaging, tractography, interhemispheric asymmetry, neuropsychological assessment, emotional symptoms, personalized rehabilitation, precision medicine

## Abstract

**Background:** Acquired brain injury (ABI) often produces heterogeneous cognitive and emotional outcomes that are not fully explained by conventional neuropsychological testing. Diffusion tensor imaging (DTI) tractography may capture patient-specific patterns of white matter connectivity and thereby complement clinical assessment. **Methods:** We conducted an exploratory case series of nine patients in the subacute phase of ABI (traumatic brain injury or subarachnoid hemorrhage). Each underwent a brief cognitive-emotional battery and 1.5 T DTI with deterministic tractography of major association tracts and the corpus callosum. Tract lateralization was quantified using the Structural Asymmetry Index (SAI), and individual profiles were compared with neuropsychological and emotional data. **Results:** Six patients met criteria for clinically significant anxiety, and four for depression, often dissociated from global cognitive screening. Tractography revealed heterogeneous asymmetry patterns, most often in the superior longitudinal fasciculus, uncinate fasciculus, and cingulum. In several cases, structural asymmetries diverged from neuropsychological findings, suggesting dissociations between behavioral testing and connectivity-based measures. **Conclusions:** Within-subject tract asymmetry analysis provided preliminary, potentially clinically relevant information not captured by tests alone. These findings indicate that individualized tractography could enrich the interpretation of cognitive and emotional profiles and help guide hypothesis generation for connectivity-informed neurorehabilitation.

## 1. Introduction

Acquired brain injury (ABI), including traumatic brain injury (TBI) and subarachnoid hemorrhage (SAH), is a leading cause of long-term disability in young and middle-aged adults. Its neurocognitive and affective sequelae are heterogeneous, compromising executive functioning, attention, memory, and emotional regulation, thereby limiting autonomy and social reintegration [1,2]. Although multidisciplinary rehabilitation programs have demonstrated efficacy, outcomes remain highly variable and difficult to predict, even among patients with apparently comparable lesions [3]. This variability has fostered growing interest in personalized medicine approaches that take into account each patient’s structural and functional characteristics [4], together with innovative interventions such as robotic-assisted training [5,6], attention-based therapies [7], and pharmacological or technology-driven innovations [8,9].

Affective symptoms are a frequent and disabling consequence of ABI. Meta-analyses estimate that approximately 30% of stroke survivors develop post-stroke depression [10]. Likewise, recent meta-analyses indicate an increased prevalence of both depression and anxiety after TBI, with estimates around 17% for anxiety and a substantially higher risk for depression [11]. Beyond populations with overt brain injury, large-scale studies have shown that reduced white-matter integrity correlates with depressive symptomatology even in otherwise healthy adults [12]. Further meta-analytic evidence indicates reduced fractional anisotropy in major depressive disorder, modulated by comorbid anxiety, age, and illness duration [13]. Similarly, altered integrity of the superior longitudinal fasciculus (SLF) and inferior longitudinal fasciculus (ILF) has been reported in social anxiety disorder compared with healthy controls [14]. These findings highlight that frontolimbic and frontoparietal pathways to affective regulation and justify their investigation in ABI.

Traditional group-based approaches, such as lesion–symptom mapping, have yielded valuable insights into the relationships between brain damage and cognition [15]. However, they fail to account for meaningful individual differences. Patients with similar neuropsychological scores may display divergent clinical profiles due to cognitive reserve, functional reorganization, or microstructural damage [16,17]. In this context, idiographic (within-subject) approaches—in which each patient serves as their own reference—enable the exploration of intra-individual variability and align with contemporary trends in precision neurorehabilitation [16]. Beyond region-centric perspectives, many post-ABI manifestations can also be interpreted within the theoretical framework of disconnection syndromes, whereby white-matter damage disrupts communication across distributed cortical and subcortical networks [18,19,20]. This framework provides a conceptual basis for the present tractography-based analysis of pathway asymmetry in relation to neuropsychological and behavioral manifestations.

Diffusion tensor imaging (DTI)-based tractography provides a practical and clinically accessible framework for this approach, enabling the characterization of association and commissural tracts at the individual level. Previous studies have demonstrated its sensitivity in detecting microstructural alterations in mild and moderate TBI and its prognostic value during the subacute phase [21]. In neuro-oncology, streamline-based segmentation yields anatomically plausible reconstructions even in structurally distorted brains, highlighting that tractographic methodology critically shapes clinical interpretation [22]. Along-tract analyses in glioma have further reported associations between white-matter integrity and preoperative neuropsychological performance, consolidating the clinical relevance of tractographic indices [23]. Collectively, these observations suggest that within-subject asymmetry analysis may offer complementary insights into cognitive and affective variability, even when psychometric assessments alone fail to capture such nuances.

From this framework, we hypothesize that interhemispheric differences in frontoparietal and frontolimbic tracts could help explain the heterogeneity observed in ABI and inform individualized rehabilitation hypotheses. For instance, asymmetries in the SLF have been associated with poorer cognitive flexibility, whereas imbalances in the uncinate fasciculus and cingulum are related to emotional dysregulation; these relationships should be interpreted as correlational rather than causal [24,25,26,27]. By integrating tractographic asymmetries with neuropsychological and affective profiles, this approach aims to bridge the gap between structural neuroimaging and practical rehabilitation planning [4,28].

The present study examines whether idiographic profiles of white matter tract asymmetry, obtained through clinical tractography in the subacute phase of ABI, may complement conventional neuropsychological assessment and provide clinically interpretable information to generate hypotheses for personalized, connectivity-informed neurorehabilitation.

## 2. Materials and Methods

### 2.1. Study Design

This was an observational case-series study adopting an idiographic design to explore individualized neuroanatomical and neuropsychological profiles in patients with acquired brain injury (ABI). The idiographic approach was selected to capture intra-individual variability in white matter connectivity and clinical presentation, in line with recent advances in precision neurorehabilitation [16]. The study was conducted at the University Hospital of Gran Canaria Dr. Negrín between August 2022 and June 2023, with approval from the institutional Ethics Committee for Research with Medicines (CEIm de Las Palmas) on 29 April 2022 (protocol code: 2022-206-1). All procedures adhered to the Declaration of Helsinki, and written informed consents were obtained from all participants.

### 2.2. Participants

Nine patients (4 men, 5 women; age range 24–64 years, M = 46.2, SD = 12.8) were recruited during the subacute post-injury phase (≤90 days; mean interval = 41.2 days, SD = 13.4). Etiologies included traumatic brain injury (TBI, *n* = 3) and subarachnoid hemorrhage (SAH, *n* = 6). Based on clinical neuroimaging reports, lesion laterality was classified descriptively as left-hemispheric in three SAH patients (Cases 2, 3, and 5), right-hemispheric in three SAH patients (Cases 4, 7, and 9), and diffuse/bilateral in all TBI cases (Cases 1, 6, and 8). Lesion classification was used only descriptively and not for inferential grouping.

Inclusion criteria were: (a) age 20–65 years, (b) confirmed ABI diagnosis, (c) ability to complete standardized cognitive and emotional testing, and (d) medical clearance for MRI. Exclusion criteria were: (a) premorbid severe cognitive impairment resulting in loss of independence in daily living; (b) major unstable systemic disease; or (c) substance abuse within the previous six months, as documented in the medical record.

### 2.3. Neuropsychological and Emotional Assessment

All patients underwent a structured neuropsychological evaluation administered by a licensed clinical neuropsychologist who was blinded to imaging data. The assessment battery included:

- Montreal Cognitive Assessment (MoCA; Spanish validation [29]) for global cognition.
- WAIS-III Digit Span (forward/backward) [30] for attention and working memory.
- Trail Making Test A and B [31] for processing speed and cognitive flexibility.
- Rey–Osterrieth Complex Figure, copy condition, interpreted with NEURONORMA norms [32] for visuoconstruction and planning.
- Hospital Anxiety and Depression Scale (HADS; Spanish validation [33]) for anxiety and depression.

This battery was designed to provide a concise but comprehensive overview of domains most frequently impaired after ABI. The MoCA was included as a widely used screening tool sensitive to global cognitive impairment across attention, orientation, executive function, and memory. Digit Span captured attentional and working memory capacity, whereas TMT-A and TMT-B assessed processing speed and set-shifting. The Rey–Osterrieth Figure measured visuoconstructive abilities, using Spanish normative data. Emotional symptoms were assessed with the HADS, validated for Spanish populations, to detect clinically significant anxiety and depression. Together, this brief battery combined validated tools with high clinical utility and feasibility in a public hospital setting.

### 2.4. Neuroimaging Acquisition and Tractography

Neuroimaging was performed on a Philips Achieva 1.5 T scanner.

- Diffusion-weighted imaging (DTI): single-shot echo-planar imaging, b = 1000 s/mm^2^, 32 non-collinear diffusion directions, voxel size = 2.0 × 2.0 × 2.0 mm^3^, and one b0 image.
- Structural imaging: 3D T1-weighted isotropic sequence, voxel size = 1 mm^3^.

Tractography was performed using Brainlab Elements Fibertracking v2.0 with deterministic streamline algorithms. Bilateral reconstructions included the superior longitudinal fasciculus (SLF), inferior longitudinal fasciculus (ILF), inferior and superior fronto-occipital fasciculi (IFOF and SFOF), uncinate fasciculus, and cingulum bundle. The corpus callosum was also segmented. Tracking parameters were as follows: FA threshold = 0.20, maximum turning angle = 45°, and minimum fiber length = 20 mm.

For each tract, volumetric measures were obtained and used to calculate the Structural Asymmetry Index (SAI). Asymmetry profiles were generated for each patient and compared with their neuropsychological and emotional data.

### 2.5. Structural Asymmetry Analysis

For each bilateral tract, lateralization was quantified using the Structural Asymmetry Index (SAI): SAI = (Right − Left)/((Right + Left)/2).

Positive values indicate rightward asymmetry; negative values indicate leftward predominance. For descriptive purposes, |SAI| ≥ 0.20 was considered clinically meaningful. SAI values are reported descriptively to support structural interpretation and are not used as inferential predictors.

### 2.6. Quality Control

All tract reconstructions were independently reviewed by two raters (a neuropsychologist and a neuroradiologist). Discrepancies were resolved by consensus. Reconstructions with implausible trajectories or motion artifacts were repeated following the quality control procedures described in recent reviews of diffusion MRI [34,35].

## 3. Results

### 3.1. Neuropsychological and Emotional Performance

The nine patients showed heterogeneous cognitive and emotional profiles (Table 1). MoCA scores ranged from 16 to 25 (M = 22.3, SD = 3.2); two patients (Cases 2 and 4) scored below 21, indicating possible cognitive impairment. Trail Making Test performance was variable: TMT-A ranged from 30 to 122 s, and TMT-B from 27 to 590 s. The longest TMT-B completion times were observed in Cases 2 (590 s) and 4 (292 s). WAIS-III Digit Span forward scores ranged from 3 to 9, and backward scores from 3 to 5. On the Rey–Osterrieth Complex Figure (copy), performance ranged from the 40th to the 90th percentile.

Emotional symptoms were frequent. Clinically significant anxiety (HADS-A ≥ 10) was found in 6/9 patients, and clinically significant depression (HADS-D ≥ 10) in 4/9 patients. Notably, Case 2 showed impaired global screening without affective symptoms, whereas Case 6 exhibited clinically significant anxiety and depression with global screening within normative ranges, illustrating cognitive-affective dissociations [36].

### 3.2. White Matter Tract Asymmetries

**Figure 1:**
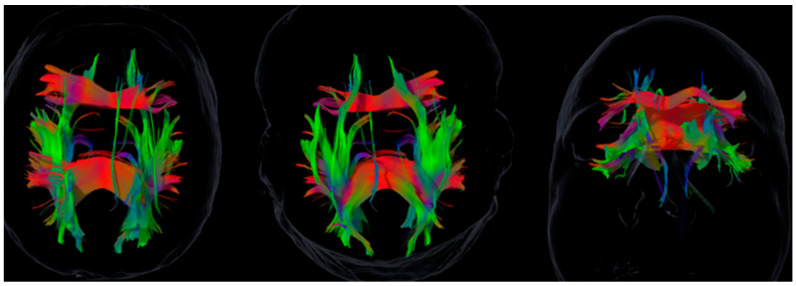
Tractography for Case 2: Left-hemisphere predominance of the superior longitudinal fasciculus (SLF). Images are shown in neurological convention (the left hemisphere appears on the left). Rendering parameters were held constant across panels. Color density indicates reconstructed streamline density (not functional magnitude). See Table 2 for tract-wise SAI values in this case.

**Figure 2:**
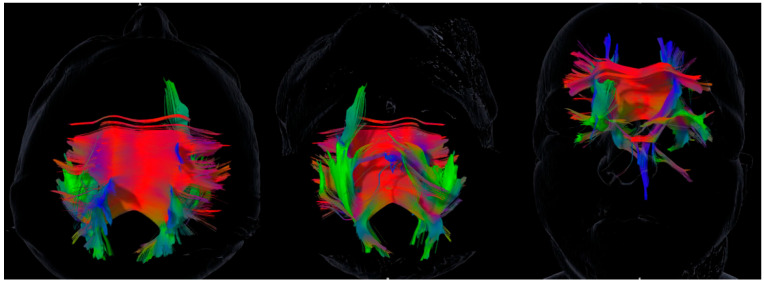
Tractography of Case 4: Predominance of the right uncinate fasciculus, and of the left superior longitudinal and cingulum bundles. Images are shown in neurological convention (the left hemisphere appears on the left). Rendering parameters were held constant across panels. Color density indicates reconstructed streamline density (not functional magnitude). See Table 2 for tract-wise SAI values in this case.

Tractography was feasible in all patients, producing individualized asymmetry profiles (Table 2, Figure 1 and Figure 2). SAI values revealed marked heterogeneity across subjects and tracts. For example, Case 4 exhibited a strongly rightward uncinate fasciculus asymmetry (SAI = +1.20) and leftward cingulum asymmetry (SAI = −0.21), whereas Case 6 showed the opposite pattern with leftward uncinate (−0.34) and rightward cingulum (+0.13).

Across the sample, clinically relevant asymmetries (|SAI| ≥ 0.20) were most frequent in the uncinate fasciculus (5/9) and the superior longitudinal fasciculus (4/9). However, asymmetry patterns did not consistently align with global screening performance: patients with comparable MoCA scores sometimes displayed divergent tract profiles, underscoring structure–function dissociations [37].

### 3.3. Integrated Profiles

Table 1 summarizes demographic, cognitive, and emotional data, while Table 2 aligns tract asymmetry indices by patient. Figure 1 and Figure 2 illustrate representative tractography, highlighting the idiographic nature of asymmetry patterns across cases and their potential relevance for clinical interpretation.

## 4. Discussion

This exploratory case series suggests that intra-subject tract asymmetry analysis may complement conventional neuropsychological assessment in patients with acquired brain injury (ABI). Despite comparable global cognitive screening scores, patients frequently displayed divergent tract profiles and dissociations between cognitive and affective symptoms. Such variability underscores the value of idiographic approaches that capture patient-specific patterns rather than assuming group-level homogeneity. Similar profiles of dissociation between cognitive and affective domains have been reported in ABI and other neurological conditions [15]. Our preliminary findings are consistent with recent proposals to develop rehabilitation strategies that explicitly integrate patient heterogeneity [16] and support ongoing efforts toward precision neurorehabilitation [23]. At the neuroanatomical level, such interindividual variability may be reflected in hemispheric asymmetries of white matter tracts.

Seminal diffusion MRI studies in healthy populations have consistently described only subtle interhemispheric asymmetries in major association tracts [18,20,26]. In contrast, the asymmetry magnitudes observed in the present sample are markedly higher, suggesting that injury-related alterations may amplify normal laterality patterns. While this observation cannot be directly compared to normative data, it highlights the potential value of individualized, within-subject analyses for characterizing structural changes after acquired brain injury.

The superior longitudinal fasciculus (SLF) was among the tracts most often showing clinically relevant asymmetries in this cohort. Previous work has associated SLF alterations with executive dysfunction and, in particular, with impaired performance on the Trail Making Test-B [38]. Likewise, uncinate fasciculus alterations have been linked to affective symptoms such as anxiety and depression [13,14], and recent studies in ABI emphasize the contribution of frontolimbic disconnections to emotional dysregulation [39]. Our observations also converge with findings implicating the cingulum bundle and related frontoparietal networks in emotional regulation and attentional control [15,16]. Taken together, these strands of evidence indicate that tract-specific asymmetry analysis could provide clinically relevant insights into executive and affective dysfunction after ABI. Nevertheless, replication in larger samples will be required to confirm these trends. Consistent with this interpretation, affective alterations were highly prevalent in our cohort.

The prevalence of affective symptoms in our sample (six patients with clinically significant anxiety and four with depression) is consistent with meta-analytic estimates of post-stroke depression [10] and the increased risk of both anxiety and depression following TBI [11].

Another contribution of this work is methodological. While group-level studies have demonstrated the prognostic and diagnostic value of diffusion tensor imaging (DTI) in TBI and SAH, few have illustrated how heterogeneity can be meaningfully addressed at the single-case level. Studies in gliomas show that along-tract analyses of white matter integrity are associated with neuropsychological outcomes [25], and tract-based biomarkers are being investigated as candidate indicators in mild TBI [17]. The present study extends this line of research by illustrating how idiographic asymmetry profiles may uncover variability that is not apparent in conventional testing. These observations align with integrative reviews highlighting the importance of combining neuroimaging, biomarkers, and rehabilitation strategies for optimized recovery after TBI [23].

From a clinical perspective, tract asymmetry profiles may serve as hypothesis-generating tools to guide individualized rehabilitation planning. For example, rightward uncinate fasciculus asymmetry associated with elevated HADS scores could justify prioritizing interventions focused on emotional regulation, whereas leftward SLF asymmetry in patients with prolonged TMT-B performance might support targeted training in cognitive flexibility. These examples are consistent with reports suggesting that individualized rehabilitation programs can improve outcomes in ABI [16] and with conceptual frameworks that advocate for the integration of idiographic neuroimaging with personalized intervention design [23].

Beyond conventional therapies, recent meta-analyses suggest that robotic-assisted interventions can improve gait, balance, and activities of daily living after stroke [5,6], while attention training programs may yield cognitive benefits in adults with moderate-to-severe TBI [7]. Likewise, umbrella reviews indicate that virtual reality interventions can enhance quality of life and daily functioning in neurological populations [21,22], and digital tools such as exergames are increasingly considered in cognitive-motor rehabilitation [9]. Pharmacological strategies, such as stimulants, have also been trialed for cognitive improvement after TBI [8].

Non-invasive brain stimulation is also gaining clinical relevance. Repetitive transcranial magnetic stimulation (rTMS) has demonstrated benefits for motor and cognitive outcomes after stroke and TBI, supported by international guidelines and recent meta-analyses [39,40]. Emerging protocols such as deep TMS and deep tDCS show preliminary evidence of feasibility in neurorehabilitation [40,41], while updated recommendations highlight the need for standardized applications [42]. These approaches are usually implemented without considering structural connectivity; integrating tractographic asymmetry analyses may help personalize stimulation strategies, although further validation is required.

### 4.1. Limitations

Several limitations must be acknowledged. The small and heterogeneous sample precludes generalization. Tractography was acquired at 1.5 T using deterministic algorithms, which are less sensitive to crossing fibers than 3T or probabilistic methods. These methodological limitations are well documented in recent reviews of diffusion MRI [34,35]. Asymmetry analyses were restricted to volumetric measures in this report, as volume is more interpretable and feasible in clinical settings. The cross-sectional design prevented evaluation of prognosis or recovery trajectories. Premorbid imaging data were not available, and pre-existing hemispheric asymmetries cannot be ruled out. This limitation is common in post-injury studies and should be addressed in future longitudinal designs. Finally, clinical correlates were restricted to a brief cognitive-emotional battery; more extensive testing and functional outcomes, such as return to work, would improve ecological validity. The neuropsychological battery focused on domains typically affected after acquired brain injury—executive functions, mood, and social-emotional regulation—using validated and widely applied instruments. Nonetheless, deficits in other areas might have gone undetected.

### 4.2. Future Directions

Future studies should aim to validate these observations in larger, prospective cohorts with longitudinal follow-up, as recommended by recent diffusion MRI reviews [34,35]. Integrating multimodal imaging modalities (structural, functional, and connectomic) and advanced diffusion metrics could increase sensitivity to subtle alterations. Additionally, broader cognitive batteries could be incorporated to capture complementary aspects of post-injury functioning. Furthermore, combining tractographic asymmetry profiles with interventional approaches such as neuromodulation or cognitive training may help determine whether idiographic connectivity patterns are associated with rehabilitation outcomes. Ultimately, such work could inform the development of clinically applicable tools within precision neurorehabilitation.

## 5. Conclusions

This exploratory case series demonstrates the feasibility of applying intra-subject tract asymmetry analysis in patients with acquired brain injury using clinically available diffusion data. Across nine cases, tract-specific asymmetries were identified in frontoparietal and frontolimbic pathways, broadly consistent with theoretical models linking these tracts to executive and affective functioning. Although the small and heterogeneous sample precludes generalization, the findings indicate that individualized tractography can complement clinical assessment by providing biologically meaningful insight consistent with current neurorehabilitation frameworks. Future studies with larger and longitudinal samples are needed to confirm these preliminary trends and to delineate their clinical significance.

## Figures and Tables

**Table 1:** Neuropsychological and emotional test scores for each patient.

Patient	Sex Male/Fem	Age	Digit Span Forward	Digit Span Backward	TMT-A (s)	TMT-B (s)	HADS-D	HADS-A	Rey Copy Type	Rey Copy Percentile	MoCA
1	M	50	5	5	30	89	11	10	50	80	25
2	M	56	5	3	122	590	5	8	50	60	20
3	M	55	9	4	30	40	6	11	10	40	25
4	F	49	6	3	59	292	0	1	25	40	16
5	F	57	6	3	30	27	4	10	75	90	23
6	M	63	5	3	45	110	17	12	25	40	24
7	F	40	5	4	41	66	9	9	75	90	24
8	F	29	3	4	30	40	11	13	75	90	22
9	F	57	4	3	69	80	11	16	10	50	22

MoCA = Montreal Cognitive Assessment (0–30); TMT = Trail Making Test; HADS-D = Hospital Anxiety and Depression Scale, Depression subscale; HADS-A = Anxiety subscale. Rey Copy Type = qualitative rating; Rey Copy Percentile = percentile for copy accuracy. Higher HADS scores indicate more severe symptoms (cut-off ≥ 10 = clinically significant). Lower TMT times reflect better performance. Pathological thresholds: MoCA < 21, HADS ≥ 10.

**Table 2:** Structural Asymmetry Index (SAI) by patient and white matter tract.

Patient	SFOF_SAI	IFOF_SAI	SLF_SAI	ILF_SAI	Unc_SAI	Cing_SAI
1	−0.037	−0.052	−0.149	−0.080	0.039	0.039
2	**−0.250**	0.003	−0.088	**−0.497**	−0.007	−0.007
3	−0.070	0.011	**0.270**	0.117	**0.221**	−0.143
4	0.094	−0.079	−0.170	0.074	**1.200**	**−0.205**
5	**0.265**	−0.011	−0.128	**0.272**	**−0.535**	0.036
6	−0.180	−0.078	**−0.348**	0.074	**−0.344**	0.127
7	0.006	−0.003	**0.223**	0.000	**−0.201**	**−0.249**
8	0.014	0.181	0.174	**0.204**	−0.063	0.061
9	−0.003	−0.065	**0.211**	−0.054	−0.013	**−0.256**

SFOF = superior fronto-occipital fasciculus; IFOF = inferior fronto-occipital fasciculus; SLF = superior longitudinal fasciculus; ILF = inferior longitudinal fasciculus. SFOF is considered a putative bundle in humans; values are reported for transparency and should be interpreted with caution. Bold indicates |SAI| ≥ 0.20.

## Data Availability

The data presented in this study are available on reasonable request from the corresponding author. The data are not publicly available due to privacy restrictions.

## Abbreviations

The following abbreviations are used in this manuscript:

ABI	Acquired Brain Injury
AD	Axial Diffusivity
CEIm	Comité de Ética de la Investigación con Medicamentos
DTI	Diffusion Tensor Imaging
HADS	Hospital Anxiety and Depression Scale
HADS-A	Hospital Anxiety and Depression Scale, Anxiety Subscale
HADS-D	Hospital Anxiety and Depression Scale, Depression Subscale
IFOF/SFOF	Fronto-Occipital Fasciculi
ILF	Inferior Longitudinal Fasciculus
MD	Mean Diffusivity
MoCA	Montreal Cognitive Assessment
MRI	Magnetic Resonance Imaging
RD	Radial Diffusivity
ROI	Region of Interest
SAH	Subarachnoid Hemorrhage
SAI	Structural Asymmetry Index
SD	Standard Deviation
SLF	Superior Longitudinal Fasciculus
SFOF	Superior Front-Occipital Fasciculus
TBI	Traumatic Brain Injury
TMT	Trail Making Test
TMT-A	Trail Making Test, Part A
TMT-B	Trail Making Test, Part B
WAIS-III	Wechsler Adult Intelligence Scale. Third Edition

## References

[1] Lu Q., Lu S., Wang X., Huang Y., Liu J., Liang Z. (2025). Structural and functional changes of post-stroke depression: A multimodal magnetic resonance imaging study. Neuroimage Clin..

[2] Stubberud J., Løvstad M., Solbakk A.-K., Schanke A.-K., Tornås S. (2020). Emotional regulation following acquired brain injury: Associations with executive functioning in daily life and symptoms of anxiety and depression. Front. Neurol..

[3] Green S.L., Gignac G.E., Watson P.A., Brosnan N., Becerra R., Pestell C., Weinborn M. (2022). Apathy and depression as predictors of activities of daily living following stroke and traumatic brain injuries in adults: A meta-analysis. Neuropsychol. Rev..

[4] Ru X., Gao L., Zhou J., Wang S., Yang J., Chen J. (2021). Secondary white matter injury and therapeutic targets after subarachnoid hemorrhage. Front. Neurol..

[5] Lee H., Kim J. (2025). Effectiveness of robot-assisted gait training in stroke rehabilitation: A systematic review and meta-analysis. J. Clin. Med..

[6] Boardsworth K., Rashid U., Olsen S., Rodríguez-Ramírez E., Browne W., Alder G., Signal N. (2025). Upper limb robotic rehabilitation following stroke: Systematic review and meta-analysis investigating efficacy and the influence of device features and program parameters. J. Neuroeng. Rehabil..

[7] Soule A.C., Fish T.J., Thomas K.G.F., Schrieff-Brown L. (2025). Attention training after moderate-to-severe traumatic brain injury in adults: A systematic review. Arch. Phys. Med. Rehabil..

[8] van der Veen R., Königs M., Bakker S., van Iperen A., Peerdeman S., Bet P.M., Oosterlaan J. (2024). Pharmacotherapy to improve cognitive functioning after Acquired brain injury: A meta-analysis and meta-regression. Clin. Pharmacol. Ther..

[9] Maggio M., Baglio F., Maione R., Calapai R., Di Iulio F., Dos Santos P.C.R., Maldonado-Díaz M., Pistorino G., Cerasa A., Quartarone A. (2025). The overlooked role of exergames in cognitive-motor neurorehabilitation. NPJ Digit. Med..

[10] Ayerbe L., Ayis S., Wolfe C.D.A., Rudd A.G. (2013). Natural history, predictors and outcomes of depression after stroke: Systematic review and meta-analysis. Br. J. Psychiatry.

[11] Ganesh A., Al-Shamli S., Mahadevan S., Chan M.F., Burke D.T., Al Rasadi K., Al Saadoon M., Al-Adawi S. (2024). The frequency of neuropsychiatric sequelae after traumatic brain injury in the Global South: A systematic review and meta-analysis. Sultan Qaboos Univ. Med. J..

[12] Nothdurfter C., Jawinski P., Markett S. (2024). White matter tract integrity is reduced in depression: Findings from the UK Biobank. Biol. Psychiatry.

[13] Xu L.M., Nguyen L., Leibenluft E., Stange J.P., Linke J.O. (2023). A meta-analysis on the uncinate fasciculus in depression. Psychol. Med..

[14] Parsaei M., Hahsemi S.M., Seyedmirzaei H., Cattarinussi G., Sambataro F., Brambilla P., Delvecchio G. (2024). Microstructural white matter alterations associated with social anxiety disorders: A systematic review. J. Affect. Disord..

[15] Forkel S.J., Friedrich P., Thiebaut de Schotten M., Howells H. (2022). White matter variability, cognition, and disorders: A systematic review. Brain Struct. Funct..

[16] Levinson C.A., Christian C., Becker C.B. (2025). How idiographic methodologies can move the clinical-science field forward to integrate personalized treatment into everyday clinical care and improve treatment outcomes. Clin. Psychol. Sci..

[17] Paolini M., Marrone S., Scalia G., Gerardi R., Bonosi L., Benigno U., Musso S., Scerrati A., Gerardo D., Signorelli F. (2025). Diffusion tensor imaging as a prognostic tool in traumatic brain injury. Brain Sci..

[18] Geschwind N. (1965). Disconnexion Syndromes in Animals and Man. Brain.

[19] Catani M., Ffytche D.H. (2005). The Rises and Falls of Disconnection Syndromes. Brain.

[20] Thiebaut de Schotten M., Foulon C., Nachev P. (2020). Brain Disconnections Link Structural Connectivity with Function (The Human Disconnectome). Nat. Commun..

[21] Hao J., Crum G., Siu K. (2024). Effects of virtual reality on stroke rehabilitation: An umbrella review. Health Sci. Rep..

[22] Olana D., Abessa T., Lamba D., Tedesco L., Bonnechere B. (2025). Effect of virtual reality-based upper limb training on activity of daily living and quality of life among stroke survivors. J. Neuroeng. Rehabil..

[23] Medina R., Dave A., Keogh C., Bartfield J., Estenssoro F., Fraga M., Lucke-Wold B. (2025). Integrating neuroimaging, biomarkers, and rehabilitation strategies for optimized diagnosis and recovery in traumatic brain injury. OBM Neurobiol..

[24] Sarubbo S., Vavassori L., Zigiotto L., Corsini F., Annicchiarico L., Rozzanigo U., Avesani P. (2024). Changing the paradigm for tractography segmentation in neurosurgery: Validation of a streamline-based approach. Brain Sci..

[25] Fahlström M., Karlsson P., Pemberton H., Sundgren P.C., Nilsson D., Orädd G., Andersson M. (2024). Qualitative and visual along-tract analysis of diffusion-based parameters in patients with diffuse gliomas. Brain Sci..

[26] Catani M., Dell’Acqua F., Thiebaut de Schotten M. (2013). A revised limbic system model for memory, emotion and behaviour. Neurosci. Biobehav. Rev..

[27] Bartolomeo P., Liu J., Seidel Malkinson T. (2025). Frontoparietal asymmetries leading to conscious perception. Trends Cogn. Sci..

[28] Pepping N., Weinborn M., Pestell C.F., Preece D.A., Malkani M., Moore S., Gross J.J., Becerra R. (2025). Improving emotion regulation ability after brain injury: A systematic review of targeted interventions. Neuropsychol. Rehabil..

[29] Pereiro A.X., Ramos-Lema S., Lojo-Seoane C., Guàrdia-Olmos J., Facal-Mayo J., Juncos-Rabadán O. (2017). Normative Data for the Montreal Cognitive Assessment (MoCA) in a Spanish Sample of Community-dweller Adults. Eur. Geriatr. Med..

[30] Wechsler D. (1999). WAIS-III: Escala de Inteligencia de Wechsler para Adultos—Manual Técnico.

[31] Arango-Lasprilla J.C., Rivera D., Aguayo A., Rodríguez W., Garza M.T., Saracho C.P., Rodríguez-Agudelo Y., Aliaga A., Weiler G., Luna M. (2015). Trail Making Test: Normative data for the Latin American Spanish speaking adult population. NeuroRehabilitation.

[32] Peña-Casanova J., Quiñones-Úbeda S., Gramunt-Fombuena N., Quintana-Aparicio M., Aguilar M., Badenes D., Molinuevo J.L., Robles A., Barquero M.S., Antúnez C. (2009). NEURONORMA Project: Norms for the Rey-Osterrieth Complex Figure Copy. Arch. Clin. Neuropsychol..

[33] Herrero M.J., Blanch J., Peri J.M., De Pablo J., Pintor L., Bulbena A. (2003). A validation study of the Hospital Anxiety and Depression Scale (HADS) in a Spanish population. Gen. Hosp. Psychiatry.

[34] Jones D.K., Knösche T.R., Turner R. (2013). White matter integrity, fiber count, and other fallacies: The do’s and don’ts of diffusion MRI. NeuroImage.

[35] Raffelt D.A., Tournier J.-D., Smith R.E., Vaughan D.N., Jackson G., Ridgway G.R., Connelly A. (2017). Investigating white matter fibre density and morphology using fixel-based analysis. NeuroImage.

[36] Murphy J.M., Bennett J.M., de la Piedad García X., Willis M.L. (2022). Emotion recognition and traumatic brain injury: A systematic review and meta-analysis. Neuropsychol. Rev..

[37] Narayana P.A. (2017). White matter changes in patients with mild traumatic brain injury: MRI perspective. Concussion.

[38] MacPherson S.E., Cox S.R., Dickie D.A., Karama S., Starr J.M., Evans C.J., Bastin M.E., Deary I.J. (2017). Processing speed and the relationship between Trail Making Test-B performance, white matter integrity, and cortical thickness in older adults. Cortex.

[39] Lefaucheur J.-P., Aleman A., Baeken C., Benninger D.H., Brunelin J., Di Lazzaro V., Filipović S.R., Grefkes C., Hasan A., Hummel F.C. (2020). Evidence-based guidelines on the therapeutic use of repetitive transcranial magnetic stimulation (rTMS): An update (2014–2018). Clin. Neurophysiol..

[40] Alashram A.R. (2025). Repetitive transcranial magnetic stimulation for cognitive rehabilitation in stroke survivors: A systematic review and meta-analysis of randomized controlled trials. Appl. Neuropsychol. Adult.

[41] Hsu W.Y., Cheng C.H., Liao K.K., Lee I.H., Lin Y.Y. (2012). Effects of repetitive transcranial magnetic stimulation on motor functions in patients with stroke: A meta-analysis. Stroke.

[42] Antal A., Alekseichuk I., Bikson M., Brockmöller J., Brunoni A., Chen R., Cohen L., Dowthwaite G., Ellrich J., Flöel A. (2017). Low intensity transcranial electric stimulation: Safety, ethical, legal regulatory and application guidelines. Clin. Neurophysiol..

